# Disrupted Resting State Network of Fibromyalgia in Theta frequency

**DOI:** 10.1038/s41598-017-18999-z

**Published:** 2018-02-01

**Authors:** Mi Kyung Choe, Manyoel Lim, June Sic Kim, Dong Soo Lee, Chun Kee Chung

**Affiliations:** 1 Department of Brain and Cognitive Sciences, College of Natural Sciences, Seoul National University, Seoul, 151-742 Republic of Korea; 2 Neuroscience Research Institute, Seoul National University College of Medicine, Seoul, 110-744 Republic of Korea; 3 Department of Neurosurgery, Seoul National University Hospital, Seoul, 110-744 Republic of Korea; 4 Department of Nuclear Medicine, Seoul National University College of Medicine, Seoul, 110-744 Korea; 5 Interdisciplinary Program in Cognitive Science, Seoul National University, Seoul, 151-742 Korea; 6 Present Address: Department of Anesthesia, Pain Management and Perioperative Medicine, Dalhousie University, Halifax, NS Canada

## Abstract

Fibromyalgia (FM), chronic widespread pain, exhibits spontaneous pain without external stimuli and is associated with altered brain activities during resting state. To understand the topological features of brain network in FM, we employed persistent homology which is a multiple scale network modeling framework not requiring thresholding. Spontaneous magnetoencephalography (MEG) activity was recorded in 19 healthy controls (HCs) and 18 FM patients. Barcode, single linkage dendrogram and single linkage matrix were generated based on the proposed modeling framework. In theta band, the slope of decrease in the number of connected components in barcodes showed steeper in HC, suggesting FM patients had decreased global connectivity. FM patients had reduced connectivity within default mode network, between middle/inferior temporal gyrus and visual cortex. The longer pain duration was correlated with reduced connectivity between inferior temporal gyrus and visual cortex. Our findings demonstrated that the aberrant resting state network could be associated with dysfunction of sensory processing in chronic pain. The spontaneous nature of FM pain may accrue to disruption of resting state network.

## Introduction

Fibromyalgia (FM) is a chronic widespread pain syndrome characterized by augmented sensitivity to painful stimuli as well as nonpainful stimuli (hyperalgesia and allodynia, respectively). Although the pathophysiology of pain in FM is not clearly understood, recent neuroimaging studies demonstrated that development and maintenance of FM are associated with augmented central nervous system processing^1^.

Chronic pain patients suffer from continuous pain in the absence of external stimulation, which is the most general characteristic of chronic pain^2^. Investigating the brain functional organization in the absence of any input (i.e. resting state) is critical in that it would disclose the fundamental aspect of spontaneous nature of chronic pain in FM patients. Neuroimaging studies have observed disruptions of resting state network (RSN) in FM and other kinds of chronic pain syndromes (i.e. chronic back pain, migraine, and complex regional pain syndrome)^3–5^. The default mode network (DMN) is the major component of RSN known to be more active at rest and reflects organization of the intrinsic brain network in chronic pain condition^6^. Recent studies have disclosed alterations of the DMN and clinical correlations between disruption of the DMN and symptoms in chronic pain syndromes^7,8^. The brain activity of DMN is known to be involved in affective aspects of pain perception.

Abnormal sensory processing of both painful and non-painful events in patients with chronic pain syndromes has been also demonstrated in several studies. Chronic pain patients showed altered brain responses to non-nociceptive sensory stimuli which were correlated with subjective pain sensitivity^9–12^. The abnormalities were associated with the deficit of pain inhibitory system and exaggerated response of afferent pathway for sensory stimuli^13^. Therefore, activities of sensory networks such as somatosensory network (SMN), auditory network (AN), and visual network (VN) at rest are also crucial in that chronic pain patients suffer from continuous pain without external stimulation. However, the precise pathways of sensory or affective system during resting state in chronic pain syndromes remain unclear.

Pain is an unpleasant experience that results from integration of bottom-up (sensory) and top-down (cognitive and emotional) modulation^14^. Default mode network (DMN) involved in emotional aspect of pain processing and diverse sensory networks related with non-stimuli processing in chronic pain is altered and related with symptoms shown by chronic pain patients. Therefore, investigating intrinsic network of DMN and sensory network is crucial. Most fMRI studies have investigated resting state network of chronic pain syndromes^8,15,16^. However, those fMRI studies have only detected very low frequency range (<0.1 Hz) fluctuations whereas magnetoencephalography (MEG) and electroencephalography (EEG) can be used to reveal brain activities from low to high frequency domains^17,18^. In previous chronic pain studies, chronic pain patients have displayed disrupted brain activates in high frequency domain (0.1 > Hz)^19^. Neuroimaging studies have generally investigated the brain resting state network in various frequency domains using EEG in patients with fibromyalgia. However, MEG has higher spatial resolution than EEG and therefore, is more suited for detecting altered cortical source activity in chronic pain. Brain oscillation study using MEG is important for understanding the relationship between brain network and pain perception in chronic pain.

Graph theoretical approach deals with analysis of topological characteristics of complex systems in network and provides the critical insights into structural and functional brain networks in complex systems^20,21^. Several network studies based on graph theory use binary networks since weighted network is difficult to interpret^20^. Most brain network studies during resting use thresholding to generate binary network, which is operator-dependent. However, arbitrary setting of threshold would induce the loss of information^22^. Persistent homology is a multiple scale network modeling framework building the brain network at every threshold, thereby inherently thresholding insensitive^23^. Investigations of brain network based on persistent homology have been conducted in neuroimaging studies^24–27^.

In this study, we employed persistent brain network homology to understand the invariant topological features of the network in FM during resting state. We used magnetoencephalography (MEG) to investigate abnormal characteristics of resting state network in FM patients within each of four frequency bands (theta, alpha, beta, and gamma). We hypothesized that resting state network in FM would be disrupted and brain regions of disrupted network would be associated with spontaneous pain of FM.

## Results

### Decreased global topological feature in fibromyalgia

**Figure 1:**
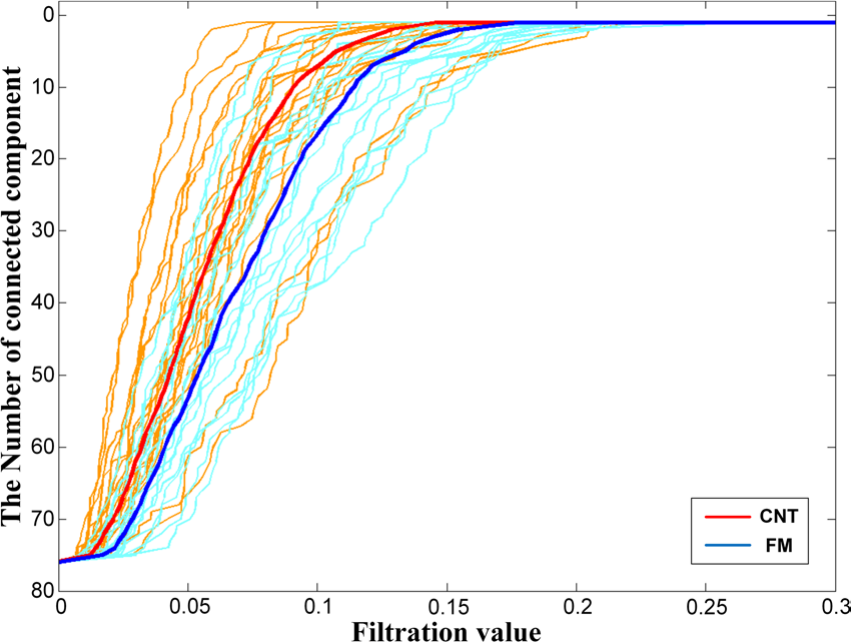
The barcode of the fibromyalgia (FM) and healthy controls (HCs) over increasing filtration values in theta frequency. Azure ones indicate that the barcodes of FM subjects, and yellow-orange ones indicate that the barcodes of HC subjects. The blue one is the mean of barcode for FM patients, and the red one is the mean of barcode for HCs. The number of connected components represents clustered set of nodes covering whole brain and indicate global connectivity of brain network.

The healthy controls (HCs) had a steeper decreasing slope of the barcode than the FM patients, which suggests that FM patients showed decreased global connectivity while the filtration value was increased in theta frequency (p < 0.05). There was no significant difference between FM patients and HCs in alpha, beta, and gamma bands (Fig. 1).

### Alterations of local network connectivity in fibromyalgia

To disclose alteration of local network properties in FM, we observed the difference in single linkage matrices (SLM) between FM patients and HCs. In theta frequency band, FM patients showed longer single linkage distance (SLD) between following pairs: left medial part of superior frontal gyrus and bilateral posterior cingulate gyrus, left precuneus and right medial part of superior frontal gyrus, left precuneus and right anterior cingulate gyrus, left anterior cingulate gyrus and bilateral posterior cingulate gyrus, left posterior cingulate gyrus and right anterior cingulate gyrus, left posterior cingulate gyrus and right precuneus, right medial part of superior frontal gyrus and right precuneus, right medial part of superior frontal gyrus and right posterior cingulate gyrus, and right anterior cingulate gyrus and right posterior cingulate gyrus. In summary, FM patients mainly had longer SLD within default mode network (DMN) regions.

**Figure 2:**
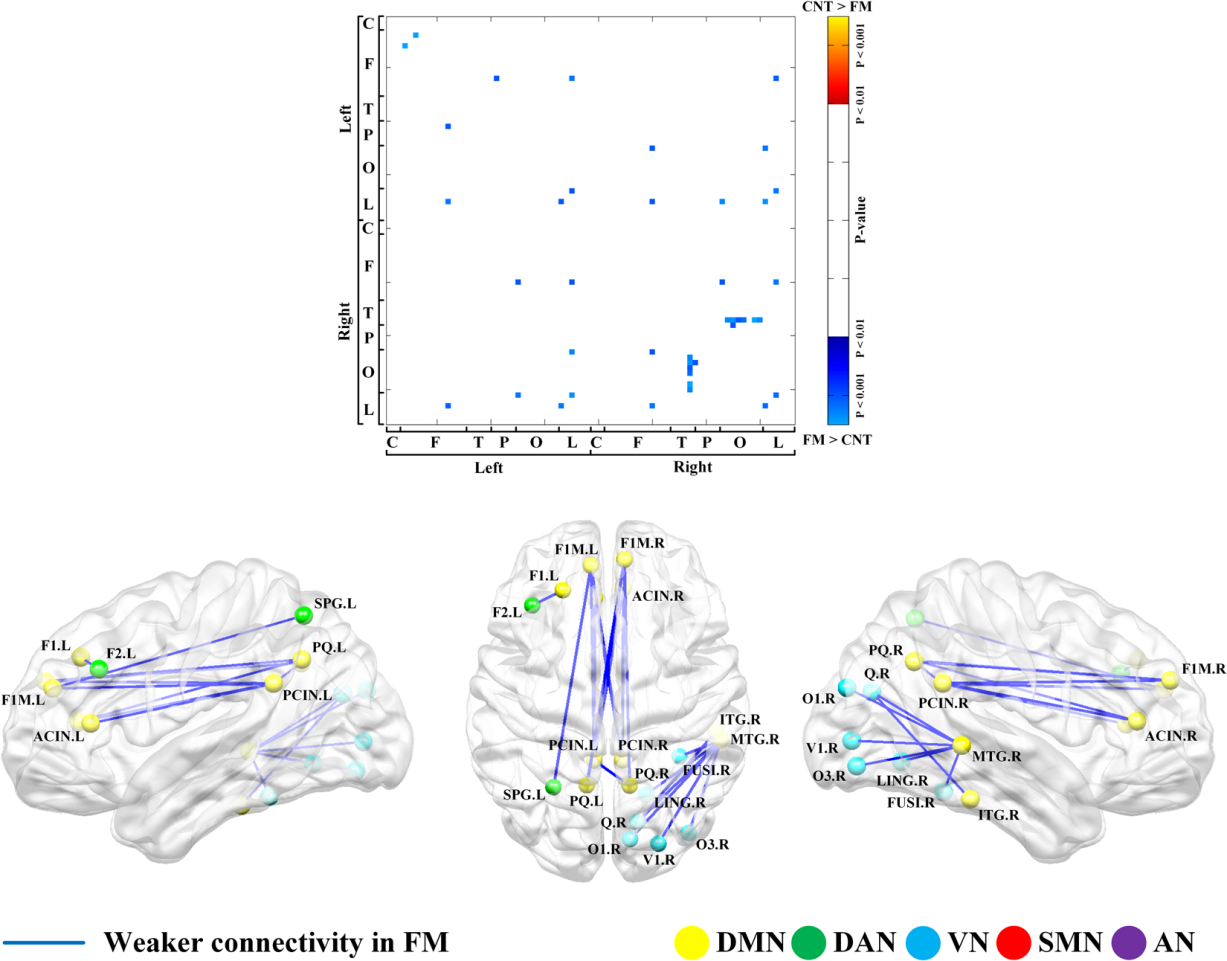
Local network properties based on persistent homology in theta band. Comparison of single linkage matrix (SLM) between fibromyalgia (FM) patients and healthy controls (HCs) (*upper*). Difference in the resting state network between FM and HC (p < 0.001) (*lower*). L, left; R, right; F1, superior frontal gyrus, dorsolateral; F2, middle frontal gyrus; F1M, superior frontal gyrus, medial; P1, superior parietal gyrus; PCIN, posterior cingulate gyrus; PQ, precuneus; ACIN, anterior cingulate gyrus; T2, middle temporal gyrus; V1, calcarine fissure; Q, cuneus; LING, lingual gyrus; O1, superior occipital gyrus; O3, Inferior occipital gyrus; FUSI, fusiform gyrus; T3, Inferior temporal gyrus; AN, auditory network; DAN, dorsal attention network; DMN, default mode network; SMN, somatosensory network; VN, visual network.

Moreover, the SLD of FM patients was longer than the SLD of HC between right middle temporal gyrus (MTG) and right visual regions (calcarine fissure, cuneus, lingual gyrus, superior/inferior occipital gyrus, and fusiform gyrus). FM group also disclosed longer SLD between right inferior temporal gyrus (ITG) and right cuneus. The SLD in FM patients was longer between left dorsolateral part of superior frontal gyrus and left middle frontal gyrus, and between medial part of left superior frontal gyrus and left superior parietal gyrus. There was no significant difference between FM patients and HCs in alpha, beta and gamma bands (Fig. 2, Supplementary Table S3).

The longer distance means later coupling during network filtration representing reduced connectivity^25^. In the FM patients, the tendency for reduced connectivity was found within the regions of default mode network in theta frequency band. Moreover, FM patients also represented decreased connectivity between MTG/ITG and visual cortex in theta band.

### Clinical symptom correlation

**Figure 3:**
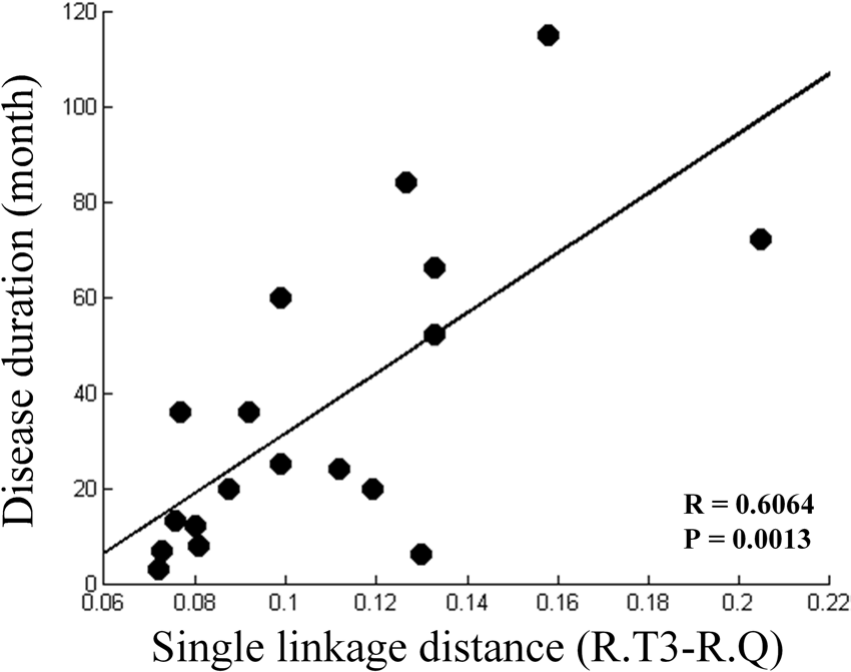
Clinical correlation between Single linkage distance (SLD) and disease duration in fibromyalgia (FM) patients. R, right; Q, cuneus; T3, Inferior temporal gyrus.

Clinical relationship between SLD and clinical symptoms were assessed. In FM group, The SLD between right inferior temporal gyrus and right cuneus showed positive correlation with disease duration of FM patients (R = 0.6064, P = 0.0013) (Fig. 3). No significant correlation between SLD and pain VAS, SF-MPQ, FIQ, BAI, or BDI was found in the FM patients.

## Discussion

In this study, we investigated brain network changes during the resting state in FM. Barcode and single linkage matrix (SLM) represent global and local characteristics of the brain network. The barcodes of FM patients displayed slower decrease in theta frequency than those of healthy controls, indicating that FM had decreased global connectivity. In theta frequency, FM patients showed longer single linkage distance (SLD) between left dorsolateral parts of superior frontal gyrus and left middle frontal gyrus. They showed longer SLD between left precuneus and medial part of left superior frontal gyrus, left precuneus and medial part of right superior frontal gyrus, left precuneus and right precuneus, left precuneus and right anterior cingulate gyrus, left posterior cingulate gyrus and medial part of right superior frontal gyrus, left posterior cingulate gyrus and right precuneus, medial part of right superior fontal gyrus and right posterior cingulate gyrus, and right anterior cingulate gyrus and right posterior cingulate gyrus. They also disclosed longer SLD between right middle temporal gyrus and right visual regions (calcarine fissure, cuneus, lingual gyrus, superior occipital cortex, and fusiform gyrus). The longer SLD between ITG and cuneus was correlated with disease duration. Overall, the result suggests that FM had reduced connectivity within DMN and sensory region. There was no significant difference between FM patients and HCs in alpha, beta, and gamma bands.

The barcode means the change in the number of connected components according to increasing single linkage distance and indicates global network feature. The present study disclosed that the barcodes of FM patients showed slower decrease in theta band. The slower decrease of the number of connected components means later network coupling, representing weaker network connectivity^25^. Therefore, the slower decrease of the barcode in FM patients suggests weaker global connectivity of resting state network in theta frequency.

The single linkage matrix (SLM) and single linkage dendrogram indicate the local network characteristics, representing which nodes could be merged. The single linkage distance (SLD) means the component of SLM and represents the functional distance of brain network^23^. In this study, the functional distance within default mode network (DMN) regions (medial prefrontal cortex, precuneus, anterior cingulate cortex, and posterior cingulate cortex) in FM patients was longer than the distance in HCs. These results disclose that FM patients showed the decreased functional connectivity of DMN. The DMN is active at rest and provides the “balance” of excitatory or inhibitory stimuli in neurons to maintain communication pathways in the brain^7,28^. Therefore, the disrupted connectivity within DMN indicates “unbalanced” of communication pathway during resting state. Then, which communication pathway become unbalanced in chronic pain? Previous neuroimaging studies exhibited aberrant connectivity among DMN regions in chronic pain syndromes^3,6,7^ and demonstrated that the disruption of DMN in chronic pain could be related to dysfunction of affective processing^7,29^.

The SLDs between middle/inferior temporal gyrus (MTG/ITG) and visual cortex (calcarine fissure, cuneus, lingual gyrus, superior occipital cortex, and fusiform gyrus) were longer in FM patients than those of HC in theta frequency. MTG participates in many other functions: MTG has been established to be parts of the visual processing with ITG and visual cortex^30,31^. MTG is also component of the default mode network. In the previous studies, the functional connectivity between visual cortex and MTG decreased in pituitary adenoma patients with visual damage^32^. In Alzheimer’s disease (AD) study, AD patients disclosed altered functional connectivity between posterior DMN and MTG and middle occipital gyrus during visual task^33^. The patients with FM showed altered brain response in visual and temporal cortex to multisensory stimulation, composed of auditory, visual, and tactile stimulation^9^. FM patients disclosed disrupted brain activities in sensory regions to non-nociceptive sensory stimuli, suggesting the significant relationship between altered responses and clinical symptoms of FM^9–12,34^. Taken together, those task studies suggest that MTG would be crucial component of the functional connectivity from visual cortex to default mode network regions as a part of processing of sensory and affective pathway.

Our result disclosed that the disrupted functional connectivity in theta frequency among DMN regions and between sensory network and MTG which is a component of sensory processing and default mode network in the absence of any other input. Theta band has been known to be involved in the wide span of cognitive function (i.e., attention, memory function, and pain perception)^35–37^. In this context, our finding of decreased functional connectivity in theta frequency within DMN would reflect “unbalanced” cognitive aspect of sensory modulation in chronic pain. MTG would be crucial region in networks of sensory and cognitive system in chronic pain syndromes.

We observed positive relationship between reduced connectivity among sensory regions and disease duration. Previous studies found altered resting state networks and suggested that ongoing pain may disrupt the brain function^7,38^. Our results indicate that the persistent pain of FM would be related to reduction of functional connectivity during resting state.

Nevertheless, there are several caveats in this study. It is of concern that this result is insufficient to suggest the specific feature only for fibromyalgia. Additional studies are necessary to reveal fibromyalgia-specific characteristic. Moreover, the participants were asked to refrain from medications three days before MEG acquisition to exclude medication effects. However, this may not be sufficient to minimize the medication effects. However, the further discontinuation of medications would be unethical. In the present study, the global and local connectivity of brain network in patients with FM were disrupted in the theta frequency. However, there were no further differences of resting state network between groups in any other frequency bands. Previous studies have reported aberrant activities of theta, alpha, beta, and gamma oscillations in chronic pain^14^. However, increase in spectral power amplitude does not necessarily imply increased connectivity^39^. Thus, it is possible that brain network difference would not be disclosed in theta, alpha, beta, and gamma frequency. The recent studies that investigated resting state network in chronic pain disclosed disruption resting state network in the theta frequency^40,41^. However, further research could improve the accuracy for investigating disrupted brain network in more segmented or more extended frequency range (i.e., beta-1, 12–16 Hz; beta-2, 16–23 Hz; beta-3, 23–30 Hz; gamma, 30–100 Hz)^42,43^. In addition, whole-brain network analysis using phase synchronization analysis is required, since the present study have used the amplitude based power correlation for network analysis in fibromyalgia.

In summary, patients with FM showed decreased functional connectivity within DMN regions and between MTG and visual cortex in theta frequency, suggesting disrupted cognitive aspect of sensory modulation and MTG, critical region in disrupted sensory modulation. They also displayed association between decreased connectivity and their ongoing pain. Our results would suggest that the aberrant network of sensory related regions in the absence of external stimulation (i.e. resting state) may be associated with pervasive sensory dysfunction in FM.

## Methods

### Participants

Eighteen right-handed female patients (age: mean = 45.1, SD = 8.5 years) were recruited from the outpatient clinics of the rheumatology departments of the Seoul National University Hospital and the Hallym University Sacred Heart Hospital. All examinations except for the diagnosis and self-report questionnaires, were conducted at the Seoul National University Hospital to ensure reliable result. Eligibility criteria for FM patients were as follows: (1) meeting the American College of Rheumatology 1990 criteria for primary FM^44^, (2) disease duration of at least 3 months, but less than 10 years, (3) score of ≥40 mm on the 0 to 100 mm pain visual analogue scale (VAS) over the previous week, (4) age between 30 and 60 years, (5) willingness to limit medications, such as, analgesics, antidepressants, and anticonvulsants, at least 3 days prior to assessments. FM patients were excluded if they had: (1) secondary FM associated with inflammatory arthritis, (2) history of substance abuse, (3) symptoms of peripheral neuropathy, (4) concomitant acute pain in the upper extremities, (5) hearing loss or use of hearing aids, (6) being pregnant or breastfeeding, (7) contraindications with MEG and MRI procedures. Nineteen healthy control subjects matched to the age and sex of FM patients (age: mean = 45.3, SD = 8.5 years) were recruited by local advertisements. The exclusion criteria for the HC group were the same as those for FM patients. The study protocol was approved by the Institutional Review Boards at Seoul National University Hospital and Hallym University Sacred Heart Hospital and was conducted in compliance with the Declaration of Helsinki. All participants in the study provided written informed consent.

### Clinical questionnaires

All participants were asked to complete clinical questionnaires. The Beck Depression Inventory (BDI)^45^, the Beck Anxiety Inventory (BAI)^46^, and the Fibromyalgia Impact Questionnaire (FIQ)^47^ were assessed. The sensory and affective components of pain were also assessed by the short-form McGill Pain Questionnaire (SF-MPQ)^48^. The resting MEG recordings and the clinical measurements of pain were acquired on the same day, excepting 9 subjects. The demographic and clinical characteristics of the subjects are displayed in Supplementary Table S1.

### MEG acquisition

The MEG signals were acquired on a VectorView™ 306-channel whole-head neuromagnetometer (Elekta Neuromag, Helsinki, Finland), organized in 204 planar gradiometers and 102 magnetometers. The participants sat comfortably beneath the helmet-shaped sensory array. They were instructed to stay relaxed and not to think about anything. Data were collected inside a magnetically shielded room for 200 seconds in an eye-closed condition. The positions of four indicator coils placed on the scalp with respect to three anatomical landmarks, the nasion and two preauricular points were measured by three-dimensional digitizer (FASTRAK, Polhemus, Colchester, VT). The x-axis of the head coordinate system passed through the two preauricular points from left to the right. The positive y-axis passed through the nasion, and the z-axis pointed up. The magnetic signals were band-pass filtered between 0.1 and 300 Hz, and were digitized at a sampling frequency of 1 kHz. The spatiotemporal signal space separation (tSSS) method using MaxFilter software (version 2.2.10; Elekta Neuromag, Helsinki, Finland) were applied to remove environmental and biological noise^49,50^.

### MRI acquisition

MRI images were acquired using a using a Magnetom TrioTim 3T scanner (Siemens, Erlangen, Germany). The following parameters were used: sagittal acquisition with a 256 × 256 matrix; field of view = 250 mm; voxel size = 1 × 1 × 1 mm; slice thickness = 1.0 mm with no gap; repetition time/echo time = 1,670/1.89 ms; flip angle = 9°; 1 excitation^50,51^.

### Data preprocessing

**Figure 4:**
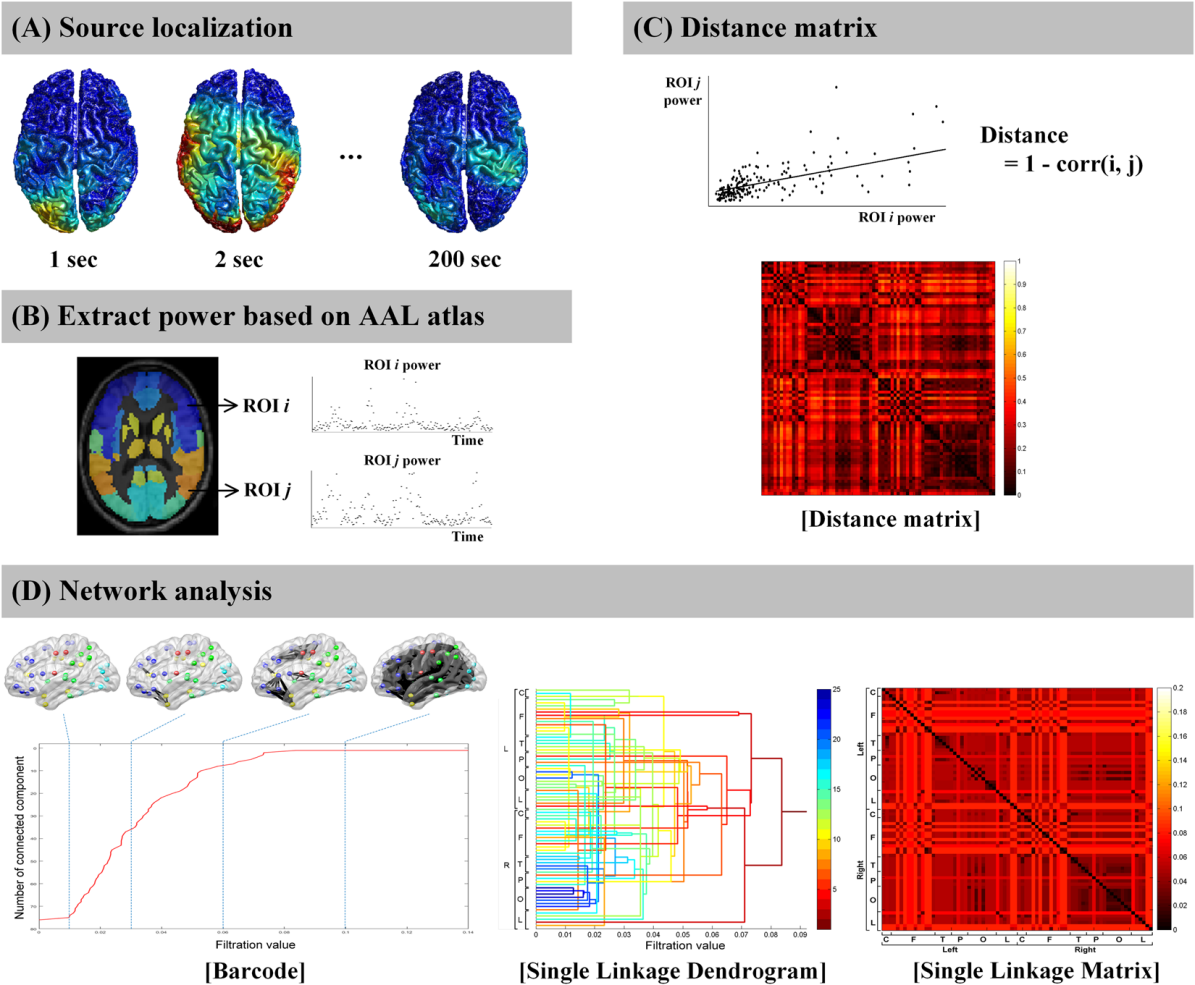
Flowchart of network analysis based on persistent homology. Neural activities on the source level were extracted at the predefined 76 nodes and the distance matrix was calculated. Graph filtration based on persistent homology was performed generating barcode, single linkage dendrogram, and single linkage matrix.

The method of brain network analysis is summarized in Fig. 4. MEG data were analyzed with MATLAB 2008b (MathWorks, Natick, MA, USA), the Fieldtrip open source software package (the Donders Institute for Brain, Cognition and Behaviour, Centre for Cognitive Neuroimaging, Nijmegen, The Netherlands, http://fieldtrip.fcdonders.nl)^52^. Prior to source analysis, independent component analysis (ICA) was applied to minimized artifacts such as electrooculography (EOG) and electrocardiogram (ECG). Continuous MEG data was split into 200 trials (1 sec per trial).

### Source analysis

The spontaneous MEG data was split into 200 epochs (1 sec per trial) without an overlap. The data for each trial (1 sec) was multitapered by Discrete Prolate Spheroidal Sequences (DPSS)^53^, a time window of 100 ms length and two tapers were applied. Each tapered data was Fourier transformed in various frequency bands: Theta (4–7 Hz), Alpha (8–12 Hz), Beta (13–30 Hz), and Gamma (31–48 Hz). Then, we averaged the power value in each epoched data, resulting in 200 averaged power values per subject and frequency domain. Beamformer analysis based on Dynamic Imaging of Coherent Sources (DICS) was performed to reconstruct neuronal source activity in the frequency domain. The Fourier transformed data for each time window, frequency, and subject was used to compute the cross spectral density^54^. The forward model was necessary to be constructed from each individual’s MRI to calculate an estimate of the field measured by the MEG sensors. A single-shell realistic description of the brain was constructed from each subject’s MRI after the structural MRI segmentation. Then, the brain volume was discretized into a grid. The lead field matrix was computed for each grid point with a 0.8 cm resolution and spatially normalized into the MNI space^52^. The spatial filter was calculated for each gird point using the cross-spectral density and the lead field matrix. Neural activity on the source level was localized by applying the filter to the Fourier transformed data.

### Network filtration based on persistent homology

#### Weighted network using interregional correlation

The 76 nodes covering whole brain except for subcortical and cerebellar regions were defined based on the Automated Anatomical Labeling (AAL) atlas (Supplementary Table S2)^55^. The AAL atlas is typically used for the brain anatomical parcellation and has been used in fMRI studies^56,57^ and MEG studies^58–60^ to obtain neuroanatomical labels of the brain network nodes. We interpolated AAL atlas onto source grid to extract source activity. Maximum power values of the whole-brain ROIs were extracted from each trial, and then trials-by-ROIs power matrices in various frequency bands (theta, alpha, beta, and gamma) were estimated per subject. To construct brain network, distance matrix was calculated through Pearson’s correlation coefficient. In other words, we denoted source activities in ROIs for 200 sec as X consisting of {*x*_1_, *x*_2_, ∙∙∙, *x*_200_}. We defined the distance *c* (*x*_*i*_, *x*_*j*_) between i-th node and j-th node by Pearson’s correlation as 1 – positive correlation coefficient. The distance matrix was calculated for each subject and frequency domain.

#### Graph filtration based on persistent homology

Based on persistent homology which is a multi-scale network modeling framework, we generated the brain network at every threshold. In the previous study^23^ which described persistent homology, a constant ɛ was defined to confine edge weights of the network to be constructed. The node *i* and *j* are connected with an edge if the distance *c* (*x*_*i*_, *x*_*j*_) between i-th node and j-th node is less than the threshold constant ɛ (i.e. *c* (*x*_*i*_, *x*_*j*_) ≤ *ɛ*). Increasing the threshold ɛ, a nested sequence of the thresholded or unweighted networks was constructed until all nodes are connected. Those multiple scale networks over the threshold are called graph filtration and the threshold distance (*ɛ*) is termed to filtration value. The topological feature over filtration is visualized by barcode and single linkage dendrogram. The barcode indicates the change in the number of connected components as the filtration values (thresholds) are increased. Regression line was calculated to define the slopes of barcode when the number of connected component was 11 to 66. The number of connected component from 11 to 66 was y value which shows nearly linear slopes among subjects in FM and HC groups. The slope of barcode was calculated for each group to estimate how fast ROIs are clustered, and it represents the global topological features of brain network. The single linkage dendrogram visualizes hierarchical clustering and shows what subnetworks could be merged during graph filtration. The dendrogram represents the local network feature in that it contains anatomical information. Single linkage matrix (SLM) is the matrix representation of the dendrogram and used for statistical group comparison. Single linkage distance (SLD) is the components of the SLM and the filtration value as nodes *i* and *j* are connected. It represents the functional distance between two brain regions.

### Statistical analysis

Statistical analysis was performed with MATLAB 2008b (MathWorks, Natick, MA, USA) and SPSS statistics 23 (Chicago, IL, USA). To compare between slopes of barcodes in FM and HC group, the independent t test was applied. Statistical significance was considered at p < 0.05. Comparing the distances of single linkage matrix, we identify the statistical difference of brain network between FM and HC. Permutation test was performed for intergroup comparisons of single linkage matrix between the groups, as follows^22^. A null hypothesis was that the single linkage distances are not different between FM and HC group. The each component in single linkage matrix (SLM) (i.e. single linkage distance (SLD)) were randomly assigned into two pseudo group matrix. The pseudo SLMs in HC and FM group were generated 10,000 times. The differences of SLDs between FM and HC group yielded a null distribution. The significant difference of SLD between FM and HC was set at uncorrected p < 0.001. Correlations between SLDs and clinical symptoms in FM patients such as SF-MPQ scores (sensory, affective, and total), current pain VAS, pain duration, and Fibromyalgia Impact Questionnaire (FIQ) were assessed using Pearson’s correlation coefficient. Linear regression analysis was performed with the single linkage distances (SLD)s between middle temporal gyrus and occipital lobe which were significantly different from those of HCs as dependent variables, and each clinical assessment as a dependent variable. P values of <0.0014 (0.05/36) were taken as statically significant after Bonferroni correction. Correlation analysis between SLDs and other symptoms in FM (BDI and BAI) was also performed. P value < 0.05 was taken as statistically significant.

## Electronic supplementary material

Supplementary Information
