# Supplementary material for: Disrupted Resting State Network of Fibromyalgia in Theta frequency
Source: Sci Rep. 2018 Feb 1;8:2064. doi: 10.1038/s41598-017-18999-z (PMC5794911; doi:10.1038/s41598-017-18999-z)
Supplement: Supplementary file 1 — Supplementary Information [file 41598_2017_18999_MOESM1_ESM.pdf]

Disrupted Resting State Network of Fibromyalgia in Theta frequency

Mi Kyung Choe[1], Manyoel Lim[2], June Sic Kim[1], and Chun Kee Chung[1,2,3,*]

[1]Department of Brain and Cognitive Sciences, College of Natural Sciences, Seoul National University, Seoul 151-742, Republic of Korea

[2]Neuroscience Research Institute, Seoul National University College of Medicine, Seoul 110-744, Republic of Korea

[3]Department of Neurosurgery, Seoul National University Hospital, Seoul 110-744, Republic of Korea

*Corresponding author: chungc@snu.ac.kr

Phone: +82-2-2072-2352

Supplementary Information

Table S1. Characteristics of the study subjects.

Variable	FM	HC	Group difference
	n = 19	n = 17	*p*-value
Demographic			
Age	45.1 (8.5)	45.3 (8.5)	0.86
Education	13.1 (2.3)	13 (2.8)	0.53
Edinburgh score	82.1 (19.9)	83 (28.1)	0.69
Clinical			
Pain duration	36.6 (31.7)	N.A.	N.A.
FIQ	62 (13.4)	N.A.	N.A.
SF-MPQ (sensory)	14.4 (6.8)	N.A.	N.A.
SF-MPQ (affective)	5.9 (2.6)	N.A.	N.A.
SF-MPQ (total)	20.3 (9)	N.A.	N.A.
Pain VAS (average past week)	61.2 (15.9)	0.5 (1.4)	< 0.001
Psychological			
BAI	23.8 (10.9)	1.4 (1.3)	< 0.001
BDI	19 (7)	2.5 (3.4)	< 0.001

All the values are expressed as mean (standard deviation), unless otherwise indicated. N.A., not applicable; FIQ, Fibromyalgia Impact Questionnaire; SF-MPQ, Short-Form McGill Pain Questionnaire; BAI, Beck Anxiety Inventory; BDI, Beck Depression Inventory.

Table S2. List of the anatomical regions of interest.

Anatomical description	Label	MNI coordinates (left)		
		x	y	z
Central				
Precentral gyrus	PRE	-43	-7	46
Rolandic operculum	RO	-44	-22	47
Postcentral gyrus	POST	-47	-5	12
Frontal				
Superior frontal gyrus, dorsolateral	F1	-22	36	38
Superior frontal gyrus, orbital part	F1O	-18	43	-18
Middle frontal gyrus	F2	-37	29	32
Middle frontal gyrus, orbital part	F2O	-34	52	-10
Inferior frontal gyrus, opercular part	F3OP	-45	13	11
Inferior frontal gyrus, triangular part	F3T	-46	29	8
Inferior frontal gyrus, orbital part	F3O	-35	29	-15
Supplementary motor area	SMA	-8	9	55
Superior frontal gyrus, medial	F1M	-8	48	25
Superior frontal gyrus, medial orbital	F1MO	-7	51	-10
Gyrus rectus	GR	-7	35	-22
Paracentral lobule	PCL	-7	-26	63
Temporal				
Insula	INS	-35	5	-1
Heschl gyrus	T2	-44	-22	9
Superior temporal gyrus	T1	-55	-20	3
Middle temporal gyrus	T2	-54	-34	-1
Inferior temporal gyrus	T3	-52	-28	-26
Parietal				
Superior parietal gyrus	P1	-26	-58	55
Inferior parietal, but supramarginal and angular gyri	P2	-45	-45	44
Supramarginal gyrus	SMG	-55	-32	29
Angular gyrus	AG	-49	-58	38
Precuneus	PQ	-10	-57	37
Occipital				
Calcarine fissure and surrounding cortex	V1	-11	-84	2
Cuneus	Q	-14	-75	23
Lingual gyrus	LING	-18	-63	-7
Superior occipital gyrus	O1	-24	-84	26
Middle occipital gyrus	O2	-39	-77	15
Inferior occipital gyrus	O3	-41	-75	-11
Fusiform gyrus	FUSI	-34	-46	-20
Limbic				

Anterior cingulate and paracingulate gyri	ACIN	-6	32	10
Median cingulate and paracingulate gyri	MCIN	-8	-16	38
Posterior cingulate gyrus	PCIN	-7	-46	27
Parahippocampal gyrus	PHIP	-26	-25	-18
Temporal pole: superior temporal gyrus	T1P	-37	10	-26
Temporal pole: middle temporal gyrus	T2P	-33	8	-40

Table S3. The significant difference of single linkage distance between FM and HC groups.

Single Linkage Distance	Region 1	Region 2	Region 1 Coordinates			Region 2 Coordinates			*p*-value
			x	y	z	x	y	z	
Theta									
FM > HC									
	L.F1	L.F2	-22	36	38	-37	29	32	0.0003
	L.F1M	L.P1	-8	48	25	-26	-58	55	0.001
	L.F1M	L.PCIN	-8	48	25	-7	-46	27	0.0006
	L.F1M	R.PCIN	-8	48	25	7	-46	27	0.0008
	L.PQ	R.F1M	-10	-57	37	8	51	28	0.0009
	L.PQ	R.ACIN	-10	-57	37	7	37	11	0.0006
	L.ACIN	L.PCIN	-6	32	10	-7	-46	27	0.001
	L.ACIN	R.PCIN	-6	32	10	7	-46	27	0.0006
	L.PCIN	R.F1M	-7	-46	27	8	51	28	0.001
	L.PCIN	R.PQ	-7	-46	27	10	-58	36	0.0005
	L.PCIN	R.ACIN	-7	-46	27	7	37	11	0.0004
	R.F1M	R.PQ	8	51	28	10	-58	36	0.001
	R.F1M	R.PCIN	8	51	28	7	-46	27	0.0006
	R.ACIN	R.PCIN	7	37	11	7	-46	27	0.0007
	R.T2	R.V1	54	-37	-1	10	-84	3	0.0005
	R.T2	R.Q	54	-37	-1	14	-75	23	0.0004
	R.T2	R.LING	54	-37	-1	18	-63	-7	0.0009
	R.T2	R.O1	54	-37	-1	24	-86	25	0.0007
	R.T2	R.O3	54	-37	-1	39	-8	-9	0.0003
	R.T2	R.FUSI	54	-37	-1	34	-45	-20	0.0005
	R.T3	R.Q	52	-33	-23	14	-75	23	0.001

FM, fibromyalgia; HC, healthy control; L, left; R, right; F1, superior frontal gyrus, dorsolateral; F2, middle frontal gyrus; F1M, superior frontal gyrus, medial; P1, Superior parietal gyrus; PCIN, posterior cingulate gyrus; PQ, precuneus; ACIN, anterior cingulate gyrus; T2, middle temporal gyrus; V1, calcarine fissure; Q, cuneus; LING, lingual gyrus; O1, superior occipital gyrus; O3, Inferior occipital gyrus; FUSI, fusiform gyrus; T3, Inferior temporal gyrus. $p < 0.001$, permutation test, uncorrected for multiple comparison.
